# Non-Communicable Neurological Disorders and Neuroinflammation

**DOI:** 10.3389/fimmu.2022.834424

**Published:** 2022-06-13

**Authors:** Clara Ballerini, Alfred K. Njamnshi, Sharon L. Juliano, Rajesh N. Kalaria, Roberto Furlan, Rufus O. Akinyemi

**Affiliations:** ^1^ Department of Experimental and Clinical Medicine, University of Florence, Florence, Italy; ^2^ Brain Research Africa Initiative (BRAIN); Neurology Department, Central Hospital Yaounde/Faculty of Medicine and Biomedical Sciences (FMBS), The University of Yaounde 1, Yaounde, Cameroon; ^3^ Neuroscience, Uniformed Services University Hebert School (USUHS), Bethesda, MD, United States; ^4^ Institute of Neuroscience, Newcastle University, Newcastle upon Tyne, United Kingdom; ^5^ Neuroscience and Ageing Research Unit, Institute for Advanced Medical Research and Training, College of Medicine, University of Ibadan, Ibadan, Nigeria; ^6^ Department of Stroke and Cerebrovascular Diseases, National Cerebral and Cardiovascular Center, Suita, Japan; ^7^ Clinical Neuroimmunology Unit, Institute of Experimental Neurology, Division of Neuroscience, Istituto di Ricerca e Cura a Carattere Scientifico (IRCCS) Ospedale San Raffaele, Milan, Italy; ^8^ Translational and Clinical Research Institute, Newcastle University, Newcastle upon Tyne, United Kingdom

**Keywords:** neuroinflammation, traumatic brain injury, stroke, alzheimer’s disease, spinal cord injury

## Abstract

Traumatic brain injury, stroke, and neurodegenerative diseases represent a major cause of morbidity and mortality in Africa, as in the rest of the world. Traumatic brain and spinal cord injuries specifically represent a leading cause of disability in the younger population. Stroke and neurodegenerative disorders predominantly target the elderly and are a major concern in Africa, since their rate of increase among the ageing is the fastest in the world. Neuroimmunology is usually not associated with non-communicable neurological disorders, as the role of neuroinflammation is not often considered when evaluating their cause and pathogenesis. However, substantial evidence indicates that neuroinflammation is extremely relevant in determining the consequences of non-communicable neurological disorders, both for its protective abilities as well as for its destructive capacity. We review here current knowledge on the contribution of neuroinflammation and neuroimmunology to the pathogenesis of traumatic injuries, stroke and neurodegenerative diseases, with a particular focus on problems that are already a major issue in Africa, like traumatic brain injury, and on emerging disorders such as dementias.

## Introduction

**Figure 1:**
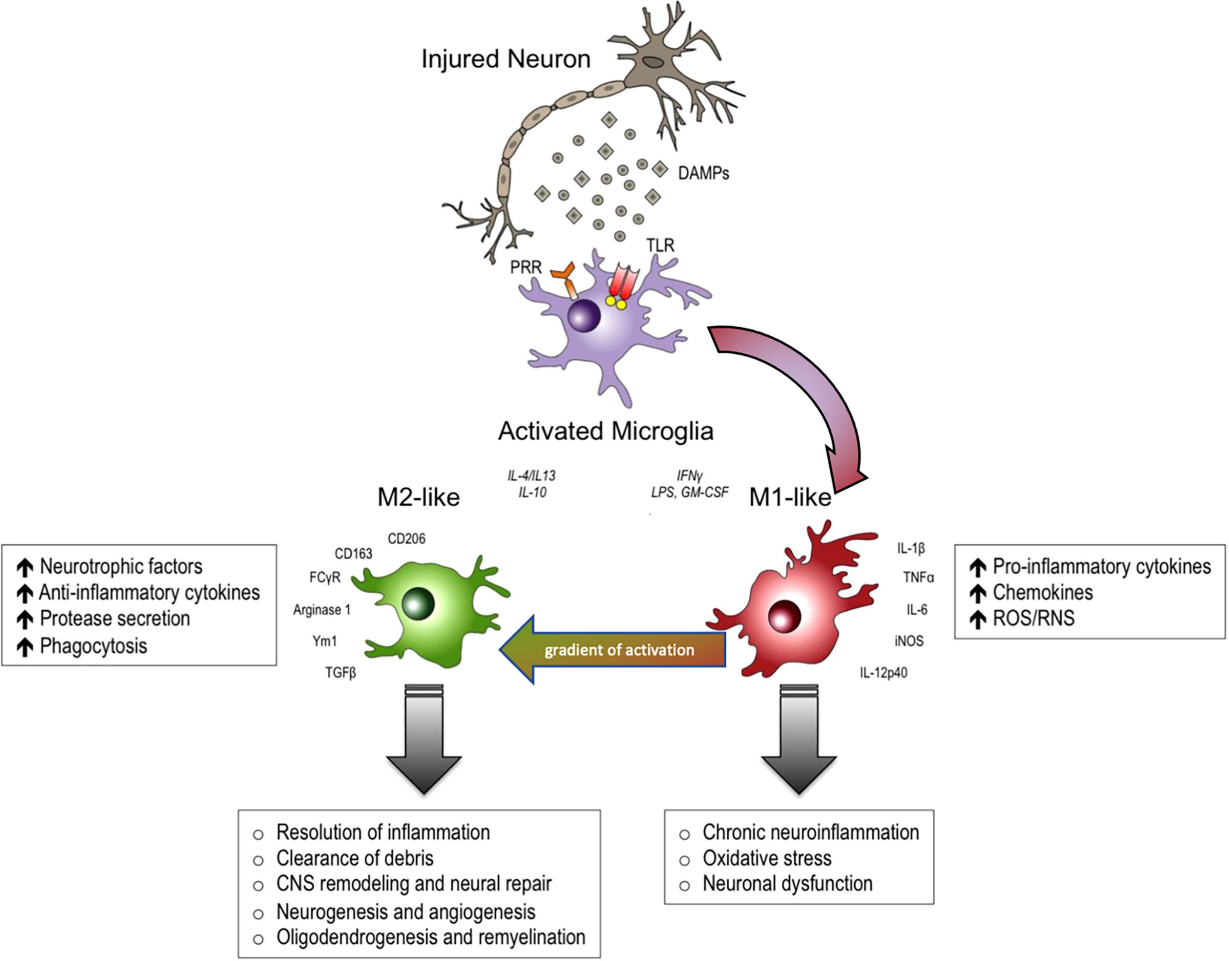
In response to danger-associated molecular patterns (DAMPs), and other extracellular signals released by injured neurons, microglia can become polarized towards pro-inflammatory M1-like and anti-inflammatory M2-like activation states that can have distinct roles in neurodegeneration and tissue repair. M1-like microglia release pro-inflammatory cytokines, chemokines and free radicals that impair brain repair and contribute to chronic neuroinflammation, oxidative stress and long-term neurological impairments. M2-like microglia release anti-inflammatory cytokines, neurotrophic factors and proteases, and they have increased phagocytic activity. M2-like microglia promote immunosuppression and resolution of M1mediated neuroinflammation, and participate in CNS remodeling and repair by modulating neurorestorative processes such as neurogenesis, angiogenesis, oligodendrogenesis and remyelineation. However, there is much overlap between and M1 and M2 like cellular responses. DAMPs, danger-associated molecular patterns; PRR, pathogen recognition receptors; TLR, toll-like receptors. Modified from (1). Reproduced with permission.

The real contribution of neuroinflammation to the pathogenesis of non-communicable diseases such as Alzheimer’s Disease, Parkinson’s Disease, amyotrophic lateral sclerosis, traumatic and spinal cord brain injury, has long been under debate, while considering both the protective and destructive effects. (1). A large number of experimental and clinical trials modulated neuroinflammation in these diseases as possible treatments, but to date we continue to only use steroids to treat brain edema after CNS injury. This is probably due to the fact that neuroinflammation is extremely heterogeneous, in terms of cells involved and their phenotype, but also heterogeneous in different brain regions (2). Despite the recent re-discovery of the significance of astrocytes, there is no doubt that microglia have been the most studied immune cell type of the CNS, and best exemplifies this diversity of functions (**Figure 1**) (1, 2). Our pharmacological tools to modulate inflammation have poor penetrance in the central nervous system (CNS) and are not able to perform the fine tuning required to re-establish the correct homeostasis of neuroinflammatory mechanisms. On the other hand, a huge knowledge gap exists concerning why neuroinflammation loses its protective functions in some pathological circumstances.

At a first glance, infectious diseases in Africa are such a relevant issue that non communicable diseases may seem a secondary problem. Africa, however, is the continent with the highest prevalence of traumatic injuries of the CNS (3), and also a region where the mean age of the population is more rapidly increasing, posing the urgent problem of treating non communicable diseases of the elderly, such as stroke and neurodegenerative disorders. We briefly review here the current knowledge on the role of neuroinflammation in these diseases, trying to focus these concepts toward the African context.

## Traumatic Brain and Spinal Cord Injuries and Neuroinflammation

Traumatic brain (TBI) and spinal cord injuries (SCI) are significantly increased in low-medium income countries of Africa, as compared to the rest of the globe (3, 4). The mean incidence is twice as high, approximatively 300/100,000 for TBI and 130 cases per million for SCI (3, 4). However, incidence distribution is not homogenous, some African nations being more affected than others (3). The major cause of TBI and SCI in Africa are road traffic accidents, accounting for 30-50% of cases, but assault and falls are also common causes (3–6). Young males are the most affected, but TBI affects all age groups, including pediatric patients, and both sexes (3). Social and ethnic disparities have also been reported (3). On the whole, despite the significant increase of publications in this field (6), reports on TBI and SCI epidemiology in African countries, are still limited in numbers, exposing a huge knowledge gap, which calls for more systematic studies.

### Pathogenesis of Traumatic Brain Injury

TBI can be classified according to different clinical scales but, based on the pathogenesis, a primary injury occurs followed by a secondary injury (7). The primary injury occurs at the moment of the concussion, and consists of tissue damage accompanied by features such as the rupture of blood vessels, neuronal damage, and haematoma. The secondary injury can last for days and involves several mechanisms, including cerebral edema and increased intracranial pressure, excitotoxicity, hypoxia, oxidative stress, and neuroinflammation (7).

Neuroinflammation secondary to TBI or SCI is primarily due to glial activation. Closed head injuries induce the production of pro-inflammatory cytokines, such as TNF-α, IL-1β, and IL-6, and of several other inflammatory mediators [reviewed in (8)], with different kinetics, peaking 1-3 days after the injury. Along with pro-inflammatory cytokines, immune-regulating and anti-inflammatory cytokines, such as IL-10 and TGF-β, become detectable in the cerebrospinal fluid with a delayed kinetic, suggesting a counterbalancing activity (8). Post-TBI neuroinflammation involves microglia and astrocytes. Microglia activate immediately after the trauma, but may last for a very long time, up to many years post TBI [reviewed in (9, 10)]. A pro-inflammatory, so-called M1 phenotype, appears first, while an anti-inflammatory, M2, phenotype appears in later stages and may last only for brief periods. Eventually after up to 5 weeks post-TBI the M1 phenotype may prevail and perpetuate damage and tissue degeneration (10). A dichotomic view has proposed also for astrocytes, by classifying them in pro-inflammatory (A1) and anti-inflammatory (A2). As for microglia, this view may be useful as a simplified model, but is largely overcome by data showing that these cells have several phenotypes according to the different stimuli received. Nevertheless, the activation of pro-inflammatory astrocytes has been reported after TBI, starting from few hours after the injury and persisting for decades (10, 11). Neuroinflammatory mechanisms contribute to clear the injured brain and spinal cord of damage and to foster tissue regenerative mechanisms, especially remyelination. The consequences of TBI and SCI, however, include cell senescence (12), that may contribute to secondary injury. While this may be the substrate for neurodegenerative mechanisms secondary to repeated head concussions, this may also explain why the severity of long-term consequences after TBI or SCI is increased in elderly (10, 12).

**Figure 2:**
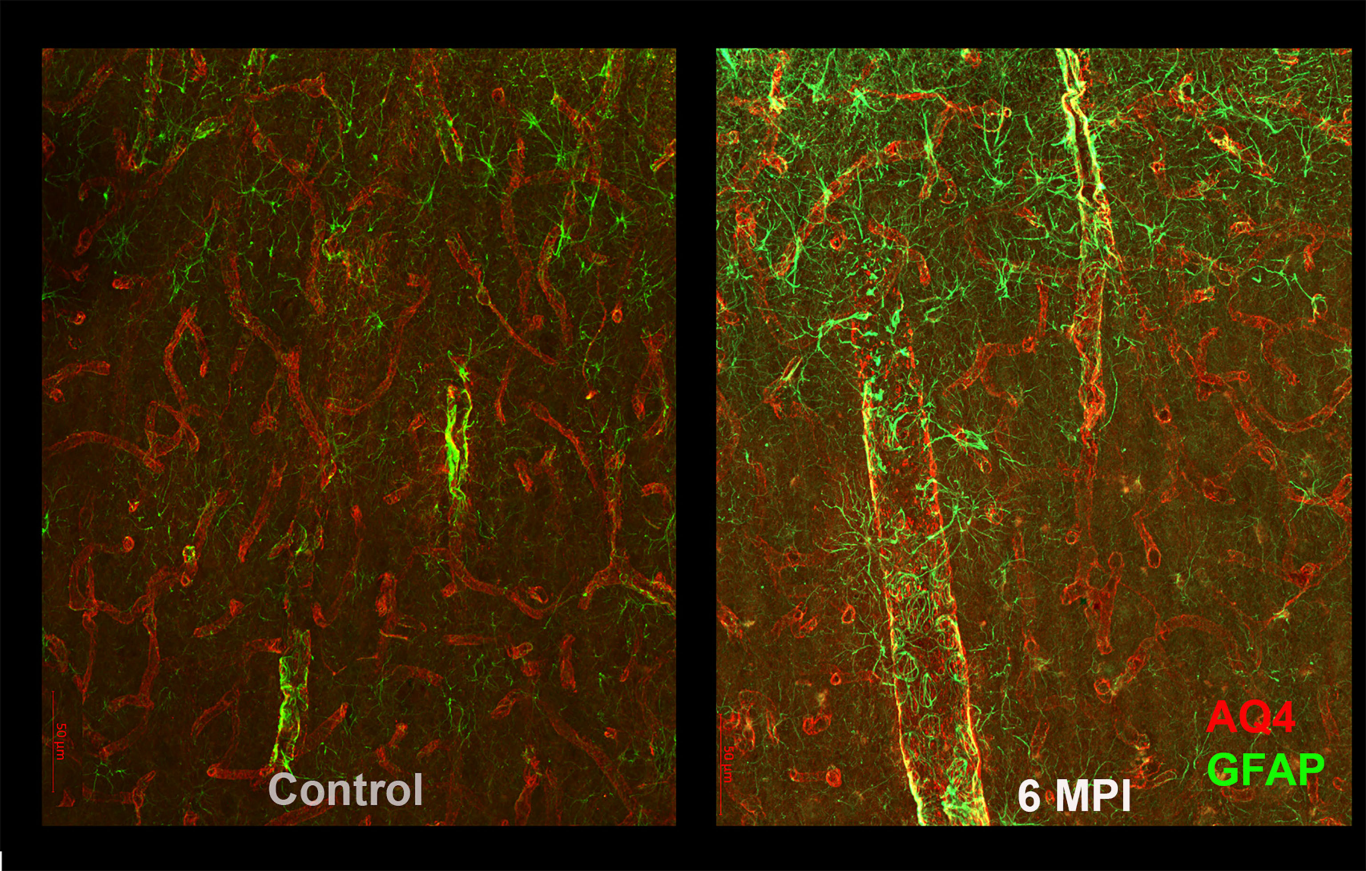
As compared to controls (right panel), reactive astrogliosis can be appreciated as an increased GFAP reactivity and morphological changes in astrocytes 6 months after brain injury. These changes are likely to affect the blood-brain barrier and the glymphatic system and thus the clearance of potentially toxic substances such as tau.

Astrocytes also play an important role in the regulation of blood flow and maintaining the blood brain barrier; in order to perform this function, they normally surround blood vessels. After TBI, in addition to generalized increased reactivity and scarring, astrocytes expand their reactivity surrounding blood vessels, and also show decreases in the polarization of their endfeet (11, 13, 14). It is likely that this change in endfeet morphology leads to impaired function in the glymphatic system, resulting in impaired clearance of toxic substances, such as tau (15) (**Figure 2**). Astrocytes also show dramatic increases that persist for many years after specific types of TBI. Perl and colleagues report that strong astrocytic scarring occurs in specific neocortical sites after blast injuries, but not other types of TBI (11).

Immune cells are implicated in the pathogenesis of TBI and SCI, but they also impact immune functions. A CNS-injury related immune deficiency has been reported, and infections are one of the leading causes of morbidity and mortality after TBI and SCI (16). Also the gut-brain axis is affected after CNS injury, and gut-dysbiosis secondary to TBI may contribute to neurodegeneration (17).

### Therapies for Neuroinflammation in TBI and SCI

Anti-inflammatory therapies or correction of gut-dysbiosis have been proposed to interfere with neuroinflammation secondary to TBI and SCI, to treat long term consequences of CNS-injury, along with several clinical trials. TBI and SCI and the underlying involvement of neuroinflammation are, however, extremely heterogeneous conditions, calling for a stratification of patients that may, or not, benefit from therapeutic intervention. An anti-inflammatory therapy targeting neuroinflammation may worsen CNS-derived immune deficiency, antibiotics to prevent infections may worsen gut dysbiosis, while dysbiosis correction always needs personalization of the intervention. There have been, however, research efforts trying to stratify patients according to peripheral inflammatory biomarkers to guide anti-IL-1β therapies (18), while integrated prognostic models to classify TBI patients in the emergency room to provide best treatment have also been validated in Africa (19). This said, steroids are largely used to treat brain and spinal cord edema secondary to traumatic injury, with all the consequences on immune system functioning.

## Stroke and Neuroinflammation

Globally, stroke remains a leading cause of dwindling economic fortunes with rapidly worsening epidemiologic indices, especially in low- and middle-income countries (LMICs) (20, 21). In low-resource settings with limited strength for acute interventional care (22), management of stroke patients is largely conservative and often the only available care. Understanding the role of neuroinflammation is sine-qua-non to improving outcomes, as inflammatory/immune response (23) in acute stroke is a major factor in stroke pathobiology (24). Inflammatory cells are involved in all the stages of acute stroke, from being potential risk factors to initial artery occlusion, brain parenchymal damage, subsequent tissue repair (25), and development of various complications (26–28). ​​Most of the complications and clinical deterioration in stroke are initiated and perpetuated by inflammatory cells and subsequent interaction with the immune system (29, 30). The ensuing “spillover-effect” leads to a systemic inflammatory response followed by immunosuppression aimed at dampening the potentially harmful proinflammatory milieu (31), thereby increasing susceptibility to post-stroke infections (16, 32, 33). This underlies the need to define the role of inflammation in the etiopathogenesis of stroke as it may offer a viable means of affordable intervention. Furthermore, stroke provides a template for the release of proinflammatory cytokines and recruitment of immune cells, which represent an important mechanism of secondary progression of brain lesion (27). Early neutrophil infiltration has been reported to be associated with larger infarct volume (34). Activated neutrophils cause the release of proteolysis enzymes such as acid phosphatase or reactive oxygen products, and exacerbate ischemic brain injury. In contrast to neutrophils, the role of different lymphocytes in acute ischemic stroke is mostly protective with release of anti-inflammatory cytokines to limit infarct size. Lymphocytes infiltrate the ischemic tissue and mediate the inflammatory response, where they increase the level of anti-inflammatory cytokines and suppress the production of proinflammatory cytokines (35). To this end, the neutrophil-lymphocyte ratio (NLR) as well as other risk markers (C-reactive protein (36, 37), Erythrocyte Sedimentation Rate, Ferritin), have been shown to be useful biomarkers of acute stroke severity and outcomes. In the longer term, particularly in patients with smaller stroke lesions, a repertoire of microglia and macrophages are recruited within the peri-infarct regions and even in subcortical grey and white matter to resolve necrotic or damaged tissue in a controlled manner (23). Current evidence suggests protective cellular mechanisms are established to repair or reseal the blood-brain barrier to prevent further damage.

Besides the role of inflammatory cells in stroke injury, it is important to recognise that the blood vessel wall undergoes inflammatory changes, which are constantly modified by age, diet, vascular and other lifestyle factors (38). Increasing age substantially reduces the inflammatory/immune response potential despite other acquired infections. In blood vessels, atherosclerosis involves significant inflammatory reactions (39) that occur during the entire process of onset, progression and rupture of atheromatous plaques (40). Among acute ischemic stroke subtypes, large artery atherosclerosis has a significantly higher inflammatory burden as determined by the NLR compared to other subtypes; low NLR positively correlated with lacunar stroke and transient ischemic attacks (TIAs) (41). A measure of inflammatory burden is now known as an emerging risk factor for incident stroke and may well predict outcomes. In the SIREN study, we found that 1 in 10 stroke cases reported antecedent history of febrile illness prior to occurrence of stroke suggesting that infectious exposures may be an important trigger of acute cerebral vascular event (42). Indeed, in previous candidate gene studies of the SIREN cohort, we demonstrated an association between the interleukin – 6 gene locus (rs1800796) and ischaemic stroke (43). Polymorphisms of the IL-6 gene regulate the circulating plasma level of interleukin – 6, a pleiotropic cytokine, which plays critical roles in the acute inflammatory response and could trigger endothelial dysfunction and activation of the coagulation – fibrinolysis system.

CNS injury may also increase susceptibility to infection. This includes stroke, which may induce immunodepression leading to secondary immunodeficiency (CNS injury-induced immunodepression [CIDS] (16) and infection. Focal cerebral ischemia induces an extensive apoptotic loss of lymphocytes and a shift from T-helper cell (Th)1 to Th2 cytokine production. Secondary lymphatic organs like the spleen (44) and thymus may also atrophy after focal cerebral ischemia thus increasing the risk of infectious complications (45) Infections (particularly chest infection and urosepsis) remain a leading cause of death in patients with stroke (46, 47) Besides having a negative effect on outcome, infection plays an important role in extending hospital stays, worsening of neurological outcomes as well as development of more serious complications, and death (48). Emerging experimental and clinical evidence strongly suggests that brain–immune interactions play an important role for outcome after stroke. These interactions may have protective, destructive, or regenerative effects in the brain, and also impact the organism as a whole (48). Risk markers that define these interactions are relevant in predicting outcome and in risk stratification.

In conclusion, further characterization and knowledge of inflammatory and immune mechanisms of stroke and the consequences that lead to vascular cognitive impairment and vascular dementia (VaD) may pave the way for an Afro-centric as well as a tailored design of new treatment to manage varying stroke subtypes (49, 50) This knowledge may also lead to management of stroke and its complications *via* modulation of immune/inflammatory response.

## Alzheimer’s Disease and Neuroinflammation

Alzheimer’s disease (AD) is a common dementing illness that manifests with progressive memory decline and cognitive dysfunction. A small fraction of familial cases, including Down syndrome patients (51), have been useful to understand pathological mechanisms and to identify heritable risk factors, although in the majority of patients, AD manifests in a sporadic form. In Africa, while there are several cases of FAD (Familial Alzheimer’s Disease), most published reports indicate the majority of AD is late-onset (LOAD) in nature (52). A review of multiple articles that included population based studies from Burkina Faso, Cameroon, Ghana, Republic of Congo, Benin Republic, Kenya, Senegal, South Africa, Central African Republic, Tanzania, and Nigeria indicates that age-adjusted prevalence of dementia varied widely ranging from 2.29% (AD 1.41%) in Nigeria-Yoruba, to 21.6% (AD prevalence not reported) in the rural Hai district of Tanzania (53). The reported prevalence of dementia for the hospital-based studies ranged from 0.05% in southwestern Nigeria to 8.87% in Dakar, Senegal. Further, 6.9% of dementia cases were found in a hospital in Tanzania and 74% in a memory clinic in South Africa. Overall, the review highlights a wide variability in the prevalence of dementia in Sub-Saharan Africa (SSA); most studies suggest a lower prevalence of dementia compared with developed countries, which may be associated with the low life expectancy in the region. In general, the authors concluded that research on the epidemiology of dementia in older persons in SSA is limited, and recent studies suggest that prevalence rates in SSA may be similar to Western countries. More recently, Akinyemi et al. showed that the burden of dementia is rising in Africa at every age (52). Prevalence varies from 2.3% to 20.0% and incidence rates are 13.3 per 1000 person-years. The most common dementia subtypes are AD, vascular dementia and human immunodeficiency virus/acquired immunodeficiency syndrome-associated neurocognitive disorders. Culture-sensitive cognitive tools not influenced by language differences are needed for implementation of more detailed studies. As indicated previously, African populations are aging and thus correlates with increased prevalence of age associated disorders of the brain as AD (51, 52). AD is considered a multifactorial disease determined by interactions between environment, lifestyle, genetics and epigenetics (54). Several studies, including Genome Wide Association Studies (GWAS), established that neuroinflammation may contribute to AD pathogenesis (55–57). Many investigations on AD animal models, mostly exploiting lipopolysaccharide (LPS) to cause brain inflammation, suggest that neuroinflammation contributes to the disease directly by increasing amyloid β (Aβ) production, although this connection is still under debate (58, 59). In general, neuroinflammation is at the same time a reaction against and a contribution to the neurodegenerative pathology in AD. Among the many factors that directly give rise to neuroinflammation in AD, microglia, a population of resident innate immune cells in the central nervous system (CNS), have been recognized as a key player. Other important elements include complement proteins, CNS infiltrating mononuclear cells, cytokines, chemokines along with other factors that may drive neuroinflammation, such as systemic inflammatory events, obesity, ageing and traumatic brain injury. Due to the recognized role of neuroinflammation in AD, cells and soluble factors participating to the inflammatory reaction may become a target of therapy and/or biomarkers of the disease. Furthermore, considering the proposed impact of peripheral chronic inflammation on AD progression (reviewed in (60)), the effects of anti-inflammatory treatments on AD have been investigated and several studies indicate that the incidence of AD is reduced in nonsteroidal anti-inflammatory users, depending on the duration of the treatment and on the presence of other risk factors (61, 62). Not all investigations confirm the protective role of non-steroidal anti-inflammatory drugs, like ibuprofen, and some even worsen inflammatory progression (63). A recent study addressing the increased risk of AD in patients affected by rheumatoid arthritis (RA) indicates that RA patients treated with anti-TNFα therapy for RA showed a reduced risk to develop AD (64).

### CNS Resident Myeloid Cell: the Multifaceted Role of Microglia in AD

AD is characterized by loss of neurons and synapses, amyloid plaques (extracellular deposition of Aβ aggregates), intraneural formation of neurofibrillary tangles, composed of hyper-phosphorylated tau (tau pathology) and neuroinflammation. Of note, proper microglia function protects brain tissue by limiting the toxic accumulation of Aβ, but in AD microglia become harmful, misbalancing normal clearance processes and promoting inflammation, mediating synapse loss and exacerbating tau pathology, that correlates with cognitive impairment (65). AD microglia, upon sensing these protein aggregates, starts a proinflammatory reaction that, due to the diffused presence of Aβ, may result in persistent inflammation. Under these conditions, microglia may shift from a protective to a harmful role and further contribute to the pathological process, accelerating the progression of neurodegeneration (66, 67). Age-related protein deposits, such as Aβ, cell debris or adenosine triphosphate, all constitute ligands that bind receptors expressed on microglia: danger –associated molecular patterns (DAMPs) and pathogen-associated molecular patterns (PAMPs) including CD36, CD14, CD47, Toll like receptors (TLRs) and NOD-like receptor family, pyrin domain-containing 3 (NRLP3) inflammosome (68–70). This activation leads to the production of cytokines (CKs), chemokines and complement (C1q), sufficient to activate astrocytes to a neurotoxic state called A1, as described in animal models of neurodegeneration and in brain tissues derived from patients. A1 astrocytes can influence the interactions of microglia with neurons and be directly harmful to neurons and synapses (71). Indeed, the release of proinflammatory cytokines such as IL-1β, IL-6, and TNFα has been detected (72–74). In the brain, these cytokines activate protein kinases in neurons and inactivate phosphatases, resulting in a further increase of tau phosphorylation and toxic self-aggregation, further amplifying the immune reaction. Altogether, the proinflammatory environment in the AD brain may directly and/or indirectly contribute to neuronal damage by several mechanisms. For example, elevated TNFα in the CSF correlates with increased rates of cognitive impairment in AD patients. In addition, inducible nitric oxide synthase, toxic to neurons, is stimulated by CKs release and upregulated in the AD brain (67).

NRLP3 is a component of the innate immune system and activated in AD brains, forming the NLP3 inflammosome complex in association with other proteins, promoting the release of proinflammatory IL-1β and IL-18. Interestingly, recent evidence suggests the NRLP3 inflammosome is also activated in VaD as a result of chronic cerebral hypoperfusion (75). Mouse models of AD deficient for NLRP3 inflammosome are protected from amyloid pathology (76) and the inflammosome in microglia plays a role in AD progression and in the spread of amyloid pathology (77). In tau-mice, after uptake and degradation, microglia are involved in generating the seeding of tau (78, 79)Tau aggregation may be linked to NLRP3 activated microglia (80), although the role of tau peptides in NLRP3 activation is still a matter of current investigation (81). Furthermore, there may be interaction between components of the autophagic pathway, (autophagy in a healthy brain contributes to maintain a heathy environment) and NLRP3-mediated neuroinflammation (82). Protein quality control and autophagy are closely related to neurodegeneration and neuroinflammation (83). Increased autophagy limits the inflammosome activity, helping cells to return to a non-reactive state; impaired autophagy activates microglia towards a proinflammatory phenotype. This is further suggested by experimentally induced deficiency of microglia autophagy, with the consequent switch of microglia to a proinflammatory state and exacerbation of tau spreading and pathology (84). Among the identified risk genes for LOAD, innate immune response genes are a category well represented, including Clusterin, TREM2 and CD33 (85). TREM2 is a cell surface receptor highly expressed in myeloid cells, including brain microglia, that stimulates phagocytosis, suppresses TLR-induced proinflammatory CKs, and contributes to the normal function of microglia in clearing Aβ deposition in the brain (86, 87). Several mutated TREM2 alleles increase the risk of AD, and the missense variant R47H, depending on the population genetic background, is a major risk factor. The R47H variant of TREM2 reduces microglia capacity of phagocytosis and of clearance of debris and apoptotic neurons, contributing to the impaired protective action of microglia and to the shift towards a proinflammatory, harmful phenotype (88). In addition, a recent work further underlines the proinflammatory effect of TREM2 genetic deficiency in the AD brain, by detecting the increased gene expression of immune networks and pathways (89).

These observations led to the possibility of identifying promising biomarkers of AD progression. Microglia respond quickly to tissue damage, therefore *in vivo* imaging of microglia cell-surface and mitochondrial membrane ligands may track inflammatory events associated with neurodegeneration. The translocator protein TSPO, increasingly expressed during neuroinflammation, is one of these targets and radiolabeled ligands to TSPO are visualized by PET, enabling detection of increased microglia activation in AD animal models and in patients. Recently Furlan et al. characterized myeloid microvesicles (MMVs) produced by activated microglia (88). MMVs are neurotoxic after loading Aβ and freshly isolated MMVs from CSF of AD patients; they are also associated with white matter damage and mild cognitive impairment (90). Investigations on these biomarkers confirm the association between cortical activation of microglia and cognitive impairment and the relation between neuroinflammation and the severity of AD (91).

More recently, the role of microglia in AD has been further explored, taking into account tau seeding driven by amyloid, simultaneously investigating both Aβ and tau (92). This study used AD animal models and focused on the role of microglia in neuritic plaques associated with tau pathology. The authors reported that TREM2 depleted microglia increase tau pathology. Surprisingly, microglia repopulation also increases tau pathology in WT mice, whereas damage associated microglia (DAM) have a protective role. Therefore, the phenotypic switch of microglia, induced by several factors including TREM2, seems crucial to the final role of these resident brain cells in limiting or inducing AD pathology during Aβ plaque-mediated tau deposition and spreading.

### Innate Immunity: the Diverse Roles of Complement System to AD

The complement system, consisting of over 40 proteins in blood and other tissues, contributes to an innate and adaptive immune defense from pathogens and injury. Together with protective effects, the complement system has a plethora of roles in immune reactions, but hyper-activation of the system may participate in pathological reactions. Three different recognition pathways activate the complement system (classical, lectin and alternative pathways) and lead to activation of an enzymatic cascade in order to mediate effector functions. This cascade is independent from the activation pathway and includes opsonization, recruitment of immune cells, generation of the lytic membrane complex for targeted death of pathogens, increase of vascular permeability and cell polarization (reviewed in (93). In the brain, the main source of the C1q complement component is microglia (94). In response to complement activation, microglia mediate synapse loss in AD and trigger inflammation through the engagement of C3a and C5a receptors. Neurotoxic reactive “A1” astrocytes express complement proteins, potentially contributing, along with microglia, to complement-mediated neurotoxicity. Of note, during brain development, synapse pruning by microglia, namely the elimination of inactive synapses, involves the classic complement pathway, C1q and C3b, which are involved in this mechanism together with microglial complement receptor 3 (CR3) (95). Excessive complement dependent synaptic pruning associates with mouse models of neurologic disorders such as AD and experimental epilepsy (96, 97). CNS expression of complement proteins increases with age; it is interesting that AD-associated genes include CR1 (gene codifying complement receptor 1), which plays a role in phagocytosis, clearance of immune complexes and inhibition of complement (98). In the AD brain, C1 inhibitors are decreased together with increased level of activators, e.g. misfolded proteins, resulting in an imbalanced control of inflammation (reviewed in (99). The presence of activated complement in human brain tissues from AD patients suggests a role of complement in the inflammatory CNS environment (100); it is mainly associated with the Aβ plaques (C1q, C3 and C4) and to a lesser extent with neurofibrillary tangles and dystrophic neurites. Neuroprotection and reduction of synaptic loss has been observed in AD mice where C1q and C3 were inhibited or knocked-out. Some authors reported that inhibition of C3 results in increases of amyloid burden, indicating that this pathway may be involved in the clearance of plaques (101). The results of microglia depletion and of complement blockage in AD models are conflicting. Nevertheless, most studies suggest that blocking the complement activation pathway has a beneficial effect on AD pathology.

Several data sets suggest that complement-mediated functions may change during AD progression. Complement may initially be anti-inflammatory, since upregulation of C1q after initial tissue injury, in the absence of other complement proteins or other danger signals, enhances microglia phagocytosis while suppressing inflammation. Later on, the complement cascade is chronically activated by accumulation of Aβ and other activators in the absence of complement regulators. Finally, C5a is generated from plaques and engages microglia by inducing chemotaxis in the plaques and proinflammatory CKs production (102).

### The Body Contribution: Proinflammatory T Cells Invade AD Brain

T lymphocytes are an important part of the adaptive immune response to infection and have specific receptors for antigens (T cell receptor, TCR), distinct from the innate immune system. T cells monitor the CNS for infection and injury, but patrolling of the CNS parenchyma is limited in non pathological conditions. In the brains of AD patients, the T cell number is instead increased, and CD8^+^T observed in the AD hippocampus (103, 104). A recent work investigated T cell subsets in the peripheral blood of persons with AD and indentified a subpopulation, CD8^+^ effector memory CD45RA^+^ T cells (TEMRA), associated with mild cognitive impairment (MCI) and AD (105). These cells are CD8^+^ memory T cells that have upregulated CD45RA, and are often senescent and terminally differentiated. These authors, by comparing a cohort of AD patients to healthy individuals, found a relation between CD8^+^TEMRA cell concentration and cognitive decline. After *in vitro* stimulation with a mitogen, cells isolated from AD patients displayed an increased production of IFNγ, a proinflammatory cytokine. The analysis of brains from AD patients confirmed the presence of these cells in the hippocampus, in the proximity of neurons, Aβ agglomerates and meninges. T cells recirculate through the cerebrospinal fluid (CSF) and brain parenchyma; molecular analysis of this cell subset in the CSF of persons with MCI and AD identified a subpopulation that showed clonal expansion bearing a TCR that may recognize the same antigen. The same finding was described in patients with Parkinson’s disease, indicating that the CSF during neurodegenerative diseases is a site of T cell expansion. In AD, clonally expanded CD8^+^ T cells residing in the hippocampus express cytotoxic genes, e.g. increased granzyme levels (104). The obvious question is, which antigens recognize expanded T cells? Authors indicate that some of expanded clones were specific for Epstein-Barr virus (EBV). This finding does not indicate a direct cause-effect relation with AD, however it may indicate a link between infections with progressive neurodegeneration and rapid cognitive decline (106). Indeed, in animal models, T cell infiltration in the brain and amyloid accumulation are triggered by peripheral infections (107). Analysis of the TCR repertoire is an expanding field, and recent study of the TCR repertoire in the brain compared to the CSF and to peripheral blood has been performed in several neurological diseases (108–110) in the attempt to identify pathologically relevant clones. Therefore, these novel findings along with continuous increasing collections of TCR sequences from the brain, the CSF, and peripheral blood of neurological patients, may allow us to compare TCR antigen specificity and identify T cell subsets that induce or restrain progression of dementia. This initial characterization of proinflammatory factors releasing T cells and infiltrating AD brains close to neurons, indicates a possible contribution of these cells to tissue damage and dysfunction in AD pathogenesis, determined by the activation of adaptive responses.

During aging, the immune system goes through a reorganization known as *immunosenescence*, where a state of chronic inflammation may damage the brain. In AD patients, and other dementias, this age-related change is exacerbated, accompanied by a skewed combination of innate versus adaptive immunity. Animal studies suggest that the adaptive immune system contributes to cognitive decline (104–106, 111). In AD, many genetic risk variants associate with innate immunity and may further drive the imbalance, due to immunosenescence or to the presence of more proinflammatory immune cells, that in turn determine the overall neurodegenerative processes. Further studies are needed to have a complete understanding of how the immune system is modified in people that develop neurodegenerative diseases, compared to people aging without cognitive decline, in order to develop therapeutically promising approaches and a new understanding of how the immune system may contribute to determining dementing diseases.

## Conclusions

Non-communicable neurological disorders represent a major health care issue in Africa. In this review we summarized current knowledge for several of these diseases in the African context and highlighted the contribution of inflammatory processes to their pathogenesis. A common concern is the lack of epidemiological data for these diseases in most African countries. This knowledge gap does not allow clear evaluation of the extent of the problem on one hand, and clear planning of possible interventions on the other. We especially reviewed the neuroimmunological aspects of these diseases. Understanding the specific contribution of inflammation to degenerative processes, while characterizing both neurodegenerative disorders, post-acute phases of stroke, and TBI may elucidate possible therapeutic strategies, waiting for other, possibly more efficacious, neuroprotective approaches to be developed.

## Author Contributions

All authors listed have made a substantial, direct, and intellectual contribution to the work and approved it for publication.

## Conflict of Interest

The authors declare that the research was conducted in the absence of any commercial or financial relationships that could be construed as a potential conflict of interest.

## Publisher’s Note

All claims expressed in this article are solely those of the authors and do not necessarily represent those of their affiliated organizations, or those of the publisher, the editors and the reviewers. Any product that may be evaluated in this article, or claim that may be made by its manufacturer, is not guaranteed or endorsed by the publisher.
